#  Inflammation in the lungs of mice due to methyl methacrylate exposure

**DOI:** 10.14202/vetworld.2020.256-260

**Published:** 2020-02-11

**Authors:** Sianiwati Goenharto, I Ketut Sudiana, Sherman Salim, Elly Rusdiana, Sri Wahjuni

**Affiliations:** 1 Department of Health, Faculty of Vocational Studies, Universitas Airlangga, Surabaya 60286, East Java, Indonesia; 2 Department of Pathology Anatomy, Faculty of Medicine, Universitas Airlangga, Surabaya 60132, East Java, Indonesia; 3 Department of Prosthodontics, Faculty of Dental Medicine, Universitas Airlangga, Surabaya 60132, East Java, Indonesia

**Keywords:** inflammation, inhalation, *in silico*, methyl methacrylate

## Abstract

**Aim:**

This study aimed to predict the potential inflammation in lungs caused by exposure to methyl methacrylate (MMA; *in silico* study) and assess inflammation in lungs in response to MMA inhalation in mice (*in vivo* study).

**Materials and Methods:**

*In silico* and *in vivo* studies were performed using 24 mice divided into a control group (0 ppm MMA) and five treatment groups, which were exposed to 150 ppm MMA for 40, 80, 120, 160, and 200 min, respectively. Lung tissues were harvested and examined with a light microscope at 400×.

**Results:**

*In silico* studies confirmed the existence of one activation bond between MMA and the toll-like receptor 4 (TLR-4), namely, His 228, with a MolDock score of −43.677 kcal/mol. Microscopic examination of lungs confirmed that a greater number of inflammatory cells were found in the treatment group than in the control group and symptoms of inflammation were clearly observable after 120 min of exposure.

**Conclusion:**

Thus, inflammation occurring due to MMA interaction with TLR-4 receptors can be predicted *in silico* and exposure to 150 ppm MMA for more than 120 min can cause lung inflammation in mice.

## Introduction

Methyl methacrylate (MMA) is a colorless, strong-smelling, and volatile liquid. It is widely used in preparing acrylic-based materials and in the manufacture of skilled-trade, medical, dental, and hobby products. MMA is considered to be a material with low toxicity. Exposure in the workplace is determined according to worker tolerance to acute upper respiratory tract irritation [1]. Azhar *et al*. evaluated the levels of MMA cytotoxicity in dental laboratory technicians and did not report any significant differences between the risk and control groups [2]. Buccal micronucleus cytome (BMCyt) assay was used in this study. Other studies involving micronuclei examination of erythrocytes showed that MMA was genotoxic on day 1 of exposure, but not on day 5 [3].

Case reports and studies have demonstrated that MMA can exert various effects that can endanger health, such as irritation of the skin, mucous membranes, and eyes, stomatitis, allergic dermatitis, neuropathy, and nervous system, and fertility disorders. Of these, the greatest concern pertains to respiratory tract irritation [4-7], with reporting cases of hypersensitive pneumonitis in dental technicians due to MMA vapor inhalation [8].

MMA exposure occurs mainly through inhalation. In 2005, the World Health Organization had determined that the threshold limit for MMA exposure in the workplace is 410 mg/m^3^ or 100 ppm. This is in accordance with the Occupational Safety and Health Code, which states that the exposure limit to MMA is 100 ppm for 8 working hours.

When performing acrylic processing, the levels of MMA inhaled by workers vary. The average may not exceed the recommended limit, but the peak of exposure can reach hundreds or even thousands of ppm, although only for a limited duration. Research conducted in Serbia has shown that MMA levels in the air can reach 2.4 times the recommended limit [9]. However, measurements of MMA levels in dental laboratories are rare because the equipment capable of automatically measuring air pollution is not easily available.

Previous study [10] on the effects of MMA exposure have been generally conducted at time intervals of 4, 8, and 12 h, and at even longer periods. In these studies, workers immediately complained about MMA vapor exposure. It was found that a single exposure to MMA for 30 min can cause temporary obstruction and restriction of airways, which subsequently decreased and disappeared on the 4^th^ day. If exposure continues, respiratory problems may ensue [10].

Therefore, it is necessary to establish the effect of MMA inhalation on inflammation of the airways through the observation of changes in histopathological features following exposure of <4 h duration. In addition, the inflammation produced can be predicted through our *in silico* study.

This study aimed to predict the potential inflammation in lungs caused by MMA exposure by analyzing its interaction with toll-like receptor 4 (TLR-4) (*in silico* study). In addition, we intended to assess inflammation in mouse lungs (*in vivo* study) following MMA exposure.

## Materials and Methods

### Ethical approval

The study was conducted on experimental subjects through *in silico* and *in vivo* approaches. This study was approved by Universitas Airlangga, Faculty of Dental Medicine Health Research Ethical Clearance Commission (Certificate No: 043/HRECCFODM/V/2018).

### Study period and study location

The study was conducted from May 2018 to October 2018 in the Laboratory of the Department of Medical Biochemistry and the Laboratory of the Department of Anatomical Pathology, Faculty of Medicine, Universitas Airlangga, Surabaya, Indonesia.

### *In silico* study

*In silico* studies were conducted to predict the inflammatory effects of MMA based on its interaction with TLR-4 receptors. Initially, two-dimensional (2D) MMA images were prepared with the ChemBioOffice Ultra 12.0 program (Cambridge Soft Co.). 2D structures were converted to 3D using the ChemBio3D 12.0 program (Cambridge Soft Co.). Using this program, the form of material stereochemistry was observed. The most stable form of regulation was attained by minimizing energy using the Merck Molecular Force Field (MMFF) 94 method, stored in the SYBYL mol2 file, and this was analyzed using the Molegro Virtual Docker (MVD) 5.5 program (CLC Bio, Aarhus, Denmark). We employed an Intel Core I-7 personal computer with a 4GB RAM and 32-bit operating system for performing the above-mentioned analyses.

Interaction with receptors was also studied using the MVD program. The first step in the docking process involved downloading the TLR-4 receptor data from the Research Collaboratory for Structural Bioinformatics-Protein Data Bank (RCSB-PDB) [11]. Cavity detection was subsequently performed on the receptor structure where the ligand was bound or interacting. The next step was to study the MMA docking on the receptors, which was carried out automatically by the MVD program.

### *In vivo* study

A laboratory-based experimental study incorporating a post-test only group design using 24 male mice (*Mus musculus* from Swiss Webster, strain (Balb/c). The mice characteristics were 3-month old with a body weight of ±20-30 g and declared healthy following a physical examination by a veterinarian. Hormones in female mice are thought to affect the conditions of this study; therefore, male mice were chosen. The mice were pre-acclimatized for 1 week at 28°C before the experiment. These subjects were maintained under a 12-h light/12-h dark cycle and fed standard diet (67.2% carbohydrates, 12.7% protein, and 5.3% fat), which were 10% of the animal body weight with *ad libitum* access to water. Diet and drinking water were given *ad libitum*. The behavior and environmental conditions of the test subjects were observed both before and during the study. The samples were divided into six groups: K0: Normal control (0 ppm of MMA), P1: Exposed to 40 min of MMA, P2: 80 min, P3: 120 min, P4: 160 min, and P5: 200 min. The room for the mouse cages was prepared with free access to breathing. The mice were exposed to MMA vapor at a concentration of 150 ppm, by placing 20 cc of MMA liquid (Merck, Germany) in four Petri dishes, each containing 5 ml of MMA and allowing it to evaporate for 90 min in the room. Next, the mice in each sample group were placed in the room for 0, 40, 80, 120, 160, and 200 min for MMA exposure. The rats were sacrificed post-treatment and a biopsy of the pulmonary alveolar tissue was collected. Microscopic slides were stained with hematoxylin-eosin solution, and histopathological examination of the harvested lung tissue was performed with a microscope at 400×.

### Statistical analysis

The data were analyzed descriptively. The MolDock score of ligand against TLR-4 was counted to find the mean value. Histopathological examination of control and treatment groups was compared qualitatively through an increase in the number of inflammatory cells.

## Results

### *In silico* study

**Figure-1:**
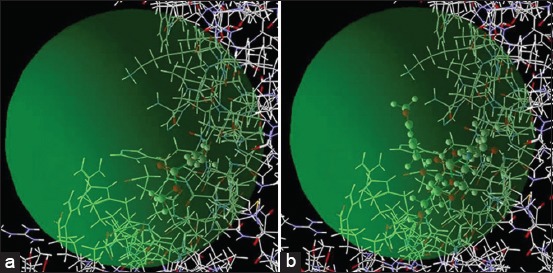
TLR 4 receptor located on the surface (GDP code: 5IJB) with: (a) A ligand NAG_701 (A), (b) MMA.

**Figure-2:**
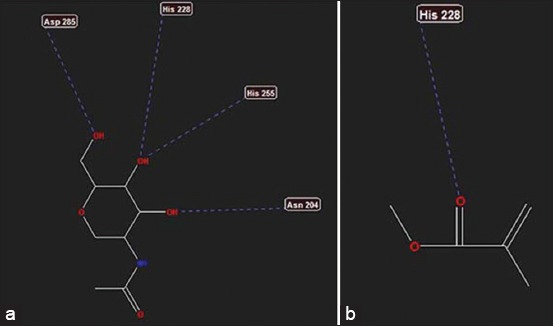
(a) Interaction of ligand NAG_701 (A) at toll-like receptor (TLR)-4, (b) interaction of methyl methacrylate at TLR-4.

This study was conducted to estimate the minimum energy from the ligand binding of MMA molecules to stimulate TLR-4. MMA and N-Acetyl-D-Glucosamine (NAG)_701 (A) ligands interacted with the surface of the TLR-4 receptors [PDB (Protein Data Bank)]) code: 5IJB; Figure-1). The interaction is shown in Figure-2.

Figure-2a and b show that there is one bond of activation from MMA to TLR-4 receptor, namely, His 228, while the ligand interaction bond is NAG_701 (A) to TLR-4, traversing through the following four steric bonds: Asp 285, His 228, His 255, and Asn 204. From Table-1, it is evident that the MolDock score is low (negative) and the MolDock NAG_701 (A) score is lower than that of MMA.

**Table-1:** MolDock score of ligand against TLR-4.

Ligan	Mean (kcal/mol)
MMA	−43.677
NAG_701(A)	−61.952

### *In vivo* study

**Figure-3:**
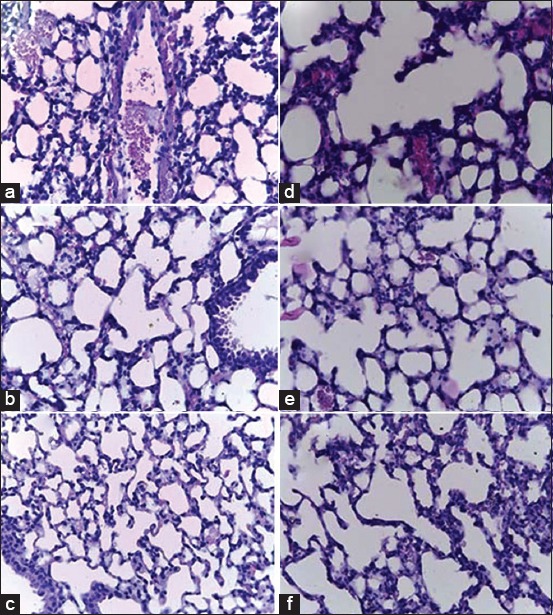
Overview of HPA on MMA exposure (a) K0: Normal control, (b) P1: Exposed to 40 min of MMA, (c) P2: 80 min, (d) P3: 120 min, (e) P4: 160 min, and (f) P5: 200 min.

Histopathological examination was conducted using a 400× light microscope, the results of which are shown in Figure-3.

Based on Figure-3, it appears that in the 40-min exposure group, the histopathological features are similar to that of the control group. After 120 min of exposure, the signs of inflammation are clearly visible through an increase in the number of inflammatory cells, namely, polymorphonuclear cells (PMN) and lymphocytes. The inflammation features were similar in the 160- and 200-min exposure groups, but lower when compared with those in the 120-min exposure group.

## Discussion

Initially, the *in silico* study was conducted on TLR-2 receptors. However, because the TLR-2 activation data in PDB remain unavailable, the study was repeated on TLR-4. The results of the *in silico* study indicated that there was one bond of activation from MMA to the TLR-4 receptor through His 228 (Figure-3). Thus, *in silico*, MMA has the potential to cause inflammation through the TLR-4 receptor. A low MolDock score (negative) indicates that the interaction of the compound or drug-receptor is stable and can be used to predict compound activity. From Table-1, it can be seen that *in silico*, both MMA and NAG_701 (A) were able to activate TLR-4 receptors, although MMA interactions were lower than those of NAG_701 (A).

Another *in silico* study of MMA structure–activity relationship (SAR), which is an analysis that employs computer methods to identify features of sub-molecular structures, showed that the methyl group on α-carbon in double acrylate bonds has decreased sensitivity and that MMA electrophilic reactions are lower than those of ester acrylic [12]. MMA has only one functional reactive group, namely, α-methyl, which substitutes double bonds; consequently, MMA is considered to be inactive and not a respiratory sensitizer [13]. Nevertheless, the role of SAR analysis for the identification of sensitivity remains limited; its use is insufficient to identify the hazardous nature of any material [14].

Alzarea *et al*. [13] reported that the toxic effects of MMA exposure could be shown through the upregulation of iNOS in the tongue tissue of rats. Although MMA is considered a potentially dangerous chemical, research into the cytotoxicity of MMA monomers using the BMCyt assay method for dental laboratory technicians wearing personal protective equipment in a well-ventilated room did not report any significant differences between the risk and control groups [2].

Studies into the effects of acute exposure to 50 ppm MMA on the upper respiratory tract have reported only a minor irritation in the nose with no other adverse effects [14]. Furthermore, there were no differences in the expression levels of IL-8 in the control and treatment groups. However, these results cannot be extrapolated to those of chronic exposure [15]. At low levels of MMA exposure, an adaptive response can occur [16].

In the present *in vivo* study, a histopathological feature was observed, which showed that exposure to MMA could increase the number of inflammatory cells in the lungs of mice. In the control group (Figure-3a), there were a minimal number of inflammatory cells present. After 40 min of exposure, the number was only slightly higher than in the control group. After 120 min of exposure, inflammatory cells were visible, especially PMN and monocytes, along with many broken alveolar septa. After 160 and 200 min of exposure, there were numerous inflammatory cells, although not as many as seen following 120 min of exposure, increased erythrocyte permeation was observed. Thus, exposure to MMA can cause inflammation in the lung tissue.

Any inflammatory response can be triggered by endogenous or exogenous stimuli. In the present case, MMA is an irritant [12]. The presence of lesions due to irritants causes active hyperemia with increased blood flow to the injured area, followed by capillary dilation and delivery of leukocytes and inflammatory cells. Neutrophils are the first leukocytes to be detected in the early stages of the inflammatory response.

Exposure to MMA acting as a free radical or reactive oxygen species (ROS) produces peroxide (O_2_), which is subsequently converted by the enzyme SOD into H_2_O_2_. Due to the activity of a catalyzing enzyme, H_2_O_2_ is converted into H_2_O and O_2_ [17]. If exposure continues, a free radical product, namely, lipid peroxidase, is formed, causing changes in cell membrane potential that, in turn, causes membrane rupture and cell death [18]. Oxidative stress in cell membranes increases malondialdehyde (MDA), a lipid membrane peroxidation product that can cause cell damage-associated molecular patterns (DAMPs). DAMPs are recognized and bound to macrophages through the surface receptor system. Consequently, in the cytosol, the signaling process begins with the activation of myeloid differentiation factor 88 (MyD88). Next, inhibitory-κB kinase (IKK) activates a phosphorylase. IKK inhibits IkB, which subsequently releases and activates NF-kB to form active NF-B, which, in turn, translocates to the nucleus to induce DNA recombination. As a result of gene transcription, translation occurs and pro-inflammatory cytokines, such as IL1, IL1β, IL-6, IL-8, and TNF-α, are produced [16].

The results obtained in this study support the theory that exposure to MMA can cause inflammation.

## Conclusion

The occurrence of inflammation in lungs due to MMA exposure can be predicted *in silico*, while histopathological features show that exposure to MMA at 150 ppm can cause lung inflammation in mice.

## Authors’ Contributions

SG contributed to the conception, research design, designed the manuscript, critically revised, and improved the manuscript. IKS contributed to the conception and research designof the manuscript. SS, ER, and SW contributed to the data acquisition and analysis and/or interpretation of data. All authors read and approved the final manuscript.
